# Exposure of the Basophilic Cell Line KU812 to Liposomes Reveals Activation Profiles Associated with Potential Anaphylactic Responses Linked to Physico-Chemical Characteristics

**DOI:** 10.3390/pharmaceutics14112470

**Published:** 2022-11-15

**Authors:** Alexander J. Plant-Hately, Burcu Eryilmaz, Christopher A. W. David, Danielle E. Brain, Bethany J. Heaton, Yvonne Perrie, Neill J. Liptrott

**Affiliations:** 1 Immunocompatibility Group, Department of Pharmacology and Therapeutics, Institute of Systems, Molecular and Integrative Biology, The University of Liverpool, Liverpool L7 3NY, UK; 2 Institute of Pharmacy and Biomedical Sciences, University of Strathclyde, Glasgow G4 0RE, UK

**Keywords:** basophil, lipidic nanoparticles, liposomes, anaphylaxis, complement activation-related pseudoallergy

## Abstract

Lipidic nanoparticles (LNP), particularly liposomes, have been proven to be a successful and versatile platform for intracellular drug delivery for decades. Whilst primarily developed for small molecule delivery, liposomes have recently undergone a renaissance due to their success in vaccination strategies, delivering nucleic acids, in the COVID-19 pandemic. As such, liposomes are increasingly being investigated for the delivery of nucleic acids, beyond mRNA, as non-viral gene delivery vectors. Although not generally considered toxic, liposomes are increasingly shown to not be immunologically inert, which may have advantages in vaccine applications but may limit their use in other conditions where immunological responses may lead to adverse events, particularly those associated with complement activation. We sought to assess a small panel of liposomes varying in a number of physico-chemical characteristics associated with complement activation and inflammatory responses, and examine how basophil-like cells may respond to them. Basophils, as well as other cell types, are involved in the anaphylactic responses to liposomes but are difficult to isolate in sufficient numbers to conduct large scale analysis. Here, we report the use of the human KU812 cell line as a surrogate for primary basophils. Multiple phenotypic markers of activation were assessed, as well as the release of histamine and inflammasome activity within the cells. We found that larger liposomes were more likely to result in KU812 activation, and that non-PEGylated liposomes were potent stimulators of inflammasome activity (four-fold greater IL-1β secretion than untreated controls), and a lower ratio of cholesterol to lipid was also associated with greater IL-1β secretion ([Cholesterol:DSPC ratio] 1:10; 0.35 pg/mL IL-1β vs. 5:10; 0.1 pg/mL). Additionally, PEGylation appeared to be associated with direct KU812 activation. These results suggest possible mechanisms related to the consequences of complement activation that may be underpinned by basophilic cells, in addition to other immune cell types. Investigation of the mechanisms behind these responses, and their impact on use in vivo, are now warranted.

## 1. Introduction

Nanoformulation of active pharmaceutical ingredients allows for a number of advantages over unformulated therapeutics, such as prolonged circulation times, altered biodistribution and reduced toxicity profiles [1,2]. Liposomal formulations have been used for some time in clinical formulations [3] and are now being increasingly investigated for the delivery of complex medicines such as proteins and nucleic acids [4]. Doxorubicin, an anthracycline drug, is widely used in chemotherapy for numerous adult and paediatric cancers but is associated with several serious side effects [5]. Doxil^®^ (Ben Venue Laboratories Inc.), a PEGylated liposomal formulation of doxorubicin and the first nano-drug to be approved by the FDA, has been successfully used to treat a number of cancers over the past few decades [1,6]. The formulation has shown to result in reduced cardiotoxicity whilst still allowing the active compound to act effectively against cancerous masses [1]. When compared to unformulated doxorubicin, it has been found that Doxil^®^ may result in more than 75% lower cardiotoxicity, along with lower reported occurrence of alopecia and nausea [7,8].

Liposomes are flexible nano-carriers, with a long history of use as drug carriers [9]. They facilitate delivery of active compounds, such as small molecule drugs and nucleic acids, by acting as a barrier between them and both the vasculature and any enzymatic systems, reducing premature degradation of the payload and prolonging blood circulation time [10,11,12,13]. However, there is a clear body of work showing that liposomes can activate the complement system which, in turn, may give rise to some of the clinical manifestations of anaphylaxis, associated with their use [14,15]. Examples of characteristics associated with liposomal activation of the complement cascade include, but are not limited to, negatively charged surfaces attracting Ca^2+^ and C1q, both being crucial to the activation of the classical pathway, ratio of cholesterol in the lipid membrane and hydrodynamic size [16,17,18,19,20,21]. Binding of complement proteins to the liposome surface, and subsequent opsonisation, has been shown to result in complement activation and inflammation, though the precise routes of this are as yet unclear and the involvement of various immune cells has been proposed [16,22]. This process of opsonization leads to an increased degree of recognition by the mononuclear phagocytic system, in particular macrophages [23,24]. The extent of the interaction with macrophages is a source of continuous intrigue, as their ability to encapsulate and transport active compounds provides increased possibilities regarding drug distribution within the body [25,26,27,28,29]. Opsonisation, by the complement system, is reduced by the addition of polyethylene glycol (PEG) to the surface and aids in this regard by providing steric repulsion to such molecules along with reducing electrostatic interactions [30]. Whilst reducing complement activation, reduced protein association also improves blood circulation half-life, via reduced damage suffered to the liposome [31].

**Figure 1:**
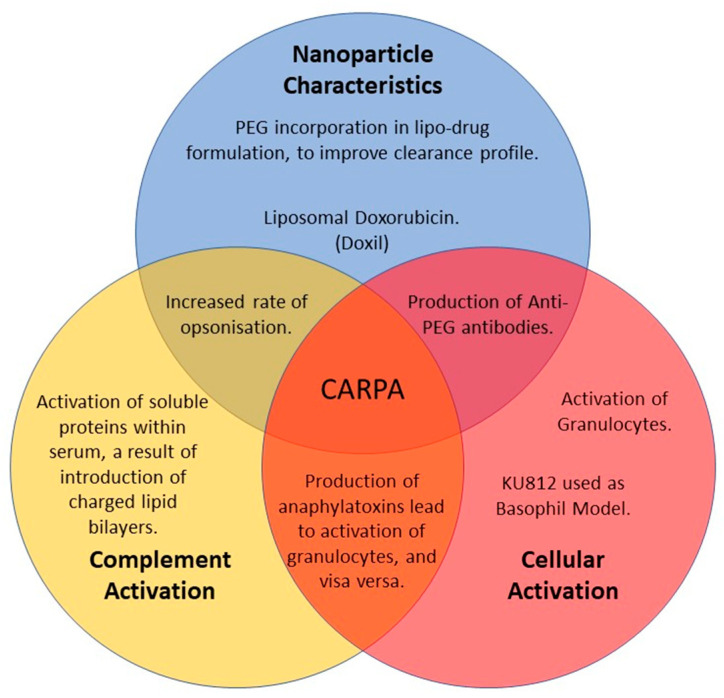
Visual representation of the multiple factors and potential contributors to incidences of CARPA.

Typically, the PEGylation of a nanocarrier leads to a significant reduction in immunogenicity compared to the administration of the active compound alone, as it reduces opsonisation and recognition by cells of the mononuclear phagocyte system (MPS) [32,33]. However, this does not remove the risk of any complement activation. PEGylation itself has been implicated in immunogenicity via pre-exposure to PEG-containing materials and the resultant generation of anti-PEG antibodies [34]. The intravenous administration of PEGylated-liposomes is linked to the occurrence of hypersensitivity reactions (HSR) [35,36]. This manifestation has been termed as complement-activation related pseudoallergy (CARPA), due to the characteristic complement activation and lack of an IgE initiated response [37]. CARPA is analogous to a typical type-I HSR (anaphylaxis), but with symptoms arising at first exposure to allergens and reducing in intensity upon repeat exposures (the inverse of type-I HSRs) [38]. The complete mechanisms responsible for CARPA are still being clarified (summarised in Figure 1), as are the physical and chemical characteristics linked to induction by PEGylated-liposomal NPs, and as such, it is difficult to determine which patients may be at greater risk of adverse events. Due to the pseudo-allergic response, related to CARPA, there is significant risk when first administering lipidic nanoparticles. The frequent occurrence of reactions (3–45%) and a lack of any reliable predeterminate testing (in vivo or in vitro), for either the patient or a novel formulation in development, has led to concern around the application of liposomes [39,40].

Human plasma and sera have been used for some time to examine the possible activation of the complement system by nano-delivery systems in general and not just liposomes [41]. However, this does not address the cellular consequences of complement activation, particularly in cells known to be involved in hypersensitivity reactions. The use of in vitro human models has been proposed to fill this void, in particular populations of basophils and mast cells, due to their key roles within complement recognition and the release in secondary mediators such as histamine [42,43,44,45]. Although they have a large degree of influence over the mechanisms of immune responses, primary basophils are difficult to obtain in sufficient numbers, due to their relatively low prevalence in peripheral blood. The KU812 cell-line has been used as a surrogate for primary basophils to enable the refined and efficient study of basophilic responses to a number of stimuli [46,47,48].

The aim of the current study was to evaluate KU812 cell responses to known anaphylatoxins and liposomal materials of varying compositions, including varying cholesterol and PEG content. In order to achieve this, KU812 populations were incubated with materials either in adequate cell-media (direct) or within pre-treated human plasma (indirect).

## 2. Materials and Methods

### 2.1. Materials

KU812 cell line were sourced from the American Type Culture Collection (ATCC). RPMI-1640 media and Foetal Bovine Serum (FBS) were purchased from Sigma Aldrich (Dorset, UK). Cobra Venom Factor (CVF) was purchased from Avanti (Alabaster, AL, USA). Antibodies for flow cytometry were purchased from Miltenyi Biotec (Bergisch Gladbach, Germany), and the MicroVue iC3b EIA was from Quidel (San Diego, CA, USA). Anaphylatoxins C3a and C5a were purchased from R & D Systems (Oxford, UK). Phorbol-12-myristate-13-acetate was purchased from Invivogen (Toulouse, France). Calcium Ionophore was purchased from Merck Life Science Limited (Dorset, UK). Used as comparators, within the study, Doxil and unloaded formulations (Doxebo) were a kind gift from Sabrina Gioria and Luigi Calzolai.

Unloaded liposome variants with varying levels of PEGylation and cholesterol: DSPC ratio, were provided by Yvonne Perrie and Burcu Eryilmaz of Strathclyde University (Scotland). These liposome variants are formulated from the same components (DSPC, DSPE-PEG2000 and cholesterol) as the Doxil/Doxebo formulations. 1,2-distearoyl-sn-glycero-3-phosphocholine (DSPC) and 1,2-distearoyl-sn-glycero-3-phosphoethanolamine-N-[methoxy (polyethylene glycol)-2000] (DSPE-PEG2000) were obtained from Lipoid (Ludwigshafen, Germany). Cholesterol (Chol) were bought from Sigma-Aldrich (St. Louis, MO, USA). Phosphate-buffered saline tablets (PBS pH 7.4) were gained from Oxoid Ltd. (Basingstoke, UK).

### 2.2. Liposome Production

Liposomes were prepared using the NanoAssemblr^®^ Benchtop from Precision NanoSystems Inc. (Vancouver, BC, Canada) which uses a Y-shape staggered herringbone micromixer. The liposomes were composed of DSPC:Cholesterol ratio of 10:1, 10:3, 10:5 weight ratio. PEGylated liposomes (DSPC: Chol: DSPE-PEG2k) were prepared with addition of 2% and 5% molarity of DSPE-PEG2k. Individual lipid stocks were prepared in ethanol and mixed to 4 mg/mL. The final lipid concentration after microfluidic production was 1 mg/mL. PBS pH 7.4 was used as aqueous phase. The production speed was 15 mL/min and the aqueous-to-organic ratio (FRR) 3:1. After production, liposomes were dialyzed against PBS pH 7.4 at RT to remove ethanol content.

### 2.3. Size Analysis of Liposomes

The size and particle size distribution of the materials was measured via the use of a zetasizer supplied by Malvern Panalytical (Malvern, UK), using the setting in Table 1. The materials in question were diluted within PBS to a final concentration of 0.1 mg/mL. The following technical parameters were used.

### 2.4. Impact of Anaphylatoxins, and Liposomes, on KU812 Proliferation and Viability via MTT and LDH Assays

KU812 cells were routinely passage in RPMI media, supplemented with 10% of foetal bovine serum (FBS). Cells were maintained aseptically in an incubator at 37 °C and (5% CO_2_). Cells were maintained at a density of 1 × 10^6^ cells/mL and prevented from exceeding 3 × 10^6^ cells/mL.

KU812 were brought to a density of 5 × 10^5^ mL^−1^ and seeded within a standard 96 well plate (100 μL per well), resulting in 5.0 × 10^4^ cells per well.

KU812 cells were incubated with various compounds, for 24 or 48 h. Concentrations used were: Anaphylatoxins C3a and C5a (6.25 nM, 12.5 nM, 25 nM, 50 nM), Phorbol-12-myristate-13-acetate and calcium ionophore (PMA/CA) (20 nM/0.5 μM, 40 nM/1 μM, 80 nM/2 μM), Doxil or Doxebo were used at serial dilutions of 200 μg/mL, 20 μg/mL, 2 μg/mL, 0.2 μg/mL, 0.02 μg/mL, 0.002 μg/mL and 0.0002 μg/mL. KU812 cells in standard media, with no compound addition, were used as untreated controls.

Samples were incubated for either 24 or 48 h at 37 °C and 5% CO_2_.

Following the incubation period, the plate was centrifuged at 2000× *g* rpm for 5 min, and the supernatant was removed and retained.

In the case of the MTT assay, each cell-containing well received 50 μL of MTT in phosphate-buffered saline (PBS) at 5 mg mL^−1^ and was incubated for a further 4 h. Resuspension within 100 μL of dimethyl sulfoxide (DMSO) was followed by absorption being measured at 570 nm and 620 nm with a calibrated Clariostar Monochromator Microplate Reader.

Conversely the LDH assay required the supernatant from each well; this was then mixed 1:1 with LDH reaction solution and incubated at 23 °C for 30 min. Absorbance was then measured at a wavelength of 490 nm, with the reference wavelength no more than 600 nm.

### 2.5. Determination of Basophil Activation in Response to Direct Stimulation

In order to observe the activation state of the basophils, it was deemed appropriate to observe the expression of CD63, CD164 and CD203c [49,50,51,52]. Flow cytometry was used to quantify the expression of each unique protein upon the cell surface. Cells at a density of 1 × 10^6^ per mL were incubated alongside treatments for either 4 or 24 h time periods. Treatments utilized throughout any investigations were as follows; untreated (negative control), PMA/CA (20 nM/0.5 μM, 40 nM/1 μM, 80 nM/2 μM), Complement 3a (6.25 nM, 12.5 nM, 25 nM, 50 nM) or Complement 5a (6.25 nM, 12.5 nM, 25 nM, 50 nM), Doxebo (200 μg/mL, 20 μg/mL, 2 μg/mL), Doxil^®^ (200 μg/mL, 20 μg/mL, 2 μg/mL) and unloaded bespoke liposomes (1000 μg/mL, 500 μg/mL, 250 μg/mL). Staining with test antibodies (AB) and isotype controls (IC), conjugated to fluorescent molecules, allowed for the level of comparison of fluorescence recorded. Data are reported as the difference in median fluorescence intensities between test antibody and isotype control. Quantification of cell-surface protein expression was performed using a multi-plex flow cytometer (MACSQuant, Bergisch Gladbach, Germany).

**Figure 2:**
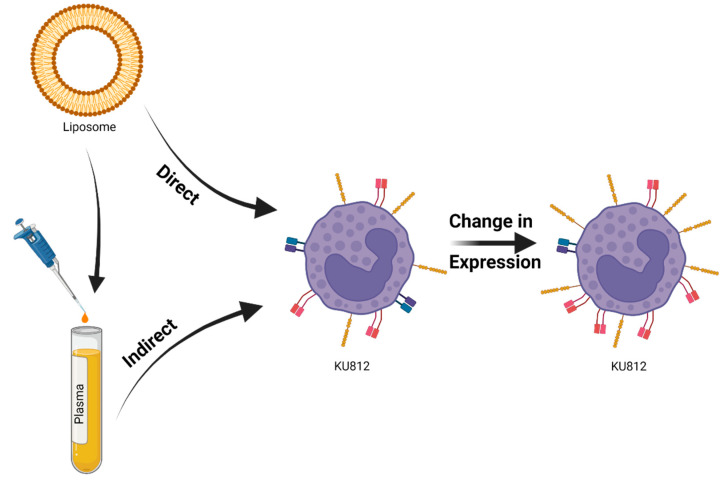
Visualisation of the routes used to expose KU812 cells within the enclosed work, through “direct” spiking of the materials onto the populations, or “indirect” treatment of an intermediate (plasma) which was then used to resuspend the KU812.

Indirect exposure of the KU812 cell-line to liposomal nanomaterials was performed via the use of healthy human plasma as an intermediary (Figure 2). Whole blood samples were extracted from three healthy volunteers, using K2EDTA as an anticoagulant. This was followed by acquisition of platelet-poor plasma through centrifuge (2500× *g*, 10 min) (Sultan, 2010). All samples were inspected to confirm absence of indicators of haemolysis, and samples exhibiting such signs were removed from testing. Remaining samples were pooled to create a uniform solution for testing. Specimens were then combined with equal measures of veronal buffer and test material (diluted in PBS). An additional control sample was also prepared which was incubated within 100% untreated plasma. They were then incubated for 30 min (37 °C and 5% CO_2_). Following this, KU812s were seeded at densities of 1 × 10^6^ cell/mL within the treated serum and incubated for a further 24 h before labelling and analysis.

### 2.6. Statistics

Results through the above investigations were expressed as mean ± SEM of *n* = 3, unless otherwise stated. Significant differences were established by (*p* < 0.05), however greater significance, if achieved, is stated. This was confirmed using one-way ANOVA or Student’s *t*-test (between two groups).

## 3. Results

Greater ratios of cholesterol to lipid, within the liposome’s composition, are associated with smaller liposome sizes.

As shown in Table 2 (below) greater cholesterol content from a ratio of 1:10 (CHOL:DSPC), to one of 5:10, was associated with a lower hydrodynamic diameter of >40% across liposomes with varying levels of PEG. In turn the initial addition of PEG (2%) to the surface of a liposome led to an increase in diameter when compared to those without (0% PEG). However, a further increase to 5% led to the reduction when compared to molecules with 2%.

**Figure 3:**
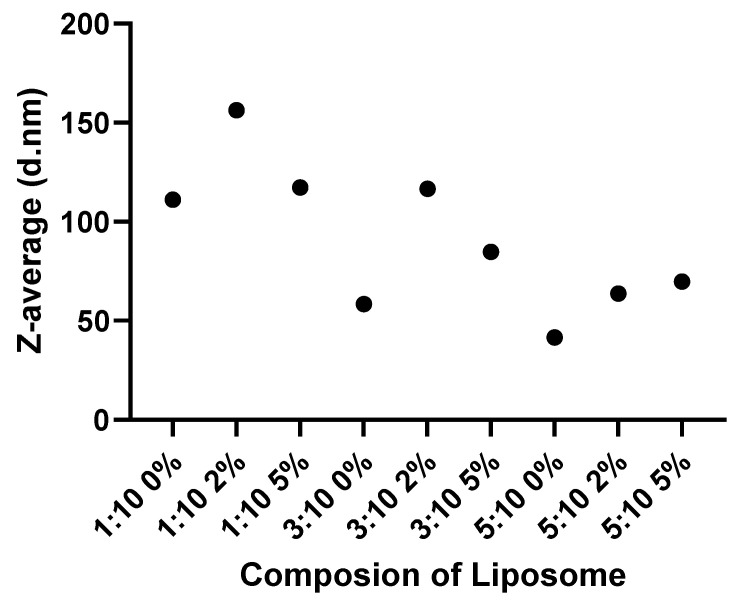
Representation of the composition-size relationship within the liposomes used. Values expressed as a mean (*n* = 3).

This relationship is further pronounced within Figure 3, with a general downward trend as the proportion of cholesterol increases. Similarly, those molecules with a middling quantity of PEG (2%) can be seen to elevate above those of 0% and 5%.

Regarding zeta-potential, we can observe the vast majority of the materials having a slight negative charge. However, it is accepted in the field that nanomaterials between −10 and 10 mV are of neutral charge [53].

### 3.1. Exposure of KU812 Cells to Anaphylatoxins Shows No Overt Impact on Proliferation, or Viability

**Figure 4:**
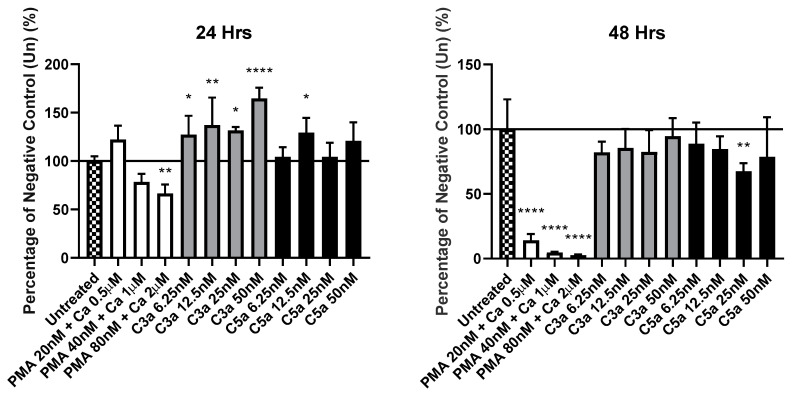
Investigation of cytotoxicity of materials via MTT assay. Level of mean absorbance measured at 570 nm, compared to that of the negative control (untreated), following 24 or 48 h incubation alongside various anaphylatoxins and stimulatory compounds. Values given are expressed as mean (*n* = 8). * denotes *p* < 0.05, ** denotes *p* < 0.01, **** denotes *p* < 0.0001. Chequered bar represents negative control (untreated), white represents conditions containing concentrations of PMA and calcium ionophore. The grey and black represent conditions of C3a and C5a respectively.

The addition of PMA and Calcium Ionophore led to a pattern of toxicity amongst KU812 cell populations, as can be seen in Figure 4. 24 h incubation shows us a significant (*p* = 0.0042) decrease in the quantity of mitochondrial activity when treated with the highest concentration (80 nM/2 µM). This is magnified after an incubation period of 48 h, with all three concentrations of PMA/CA leading to significant (*p* < 0.0001) decreases. The first of the two anaphylatoxins tested, complement 3a (C3a), led to a significant (*p* < 0.05) increase in mitochondrial activity after 24 h, however this increase was not permanent as the measured values were deemed non-significant (*p* > 0.05) after 48 h. Conversely, complement 5a (C5a) only recorded a significant (*p* < 0.05) increase at a concentration of 12.5 nM, whilst the recorded values noticeably decreased after 48 h, to the point where a treatment of 25 nM led to a significant (*p* = 0.001) decrease.

### 3.2. Impact on KU812 Viability Was Observed with Higher Concentrations of Liposomes

**Figure 5:**
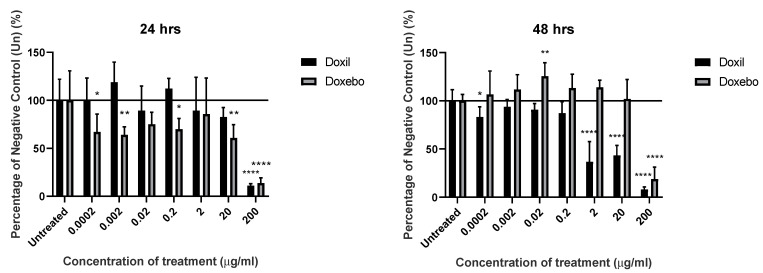
Investigation of cytotoxicity of materials via MTT assay. Level of mean absorbance measured at 570 nm, compared to that of the negative control (untreated), following 24 or 48 h incubation. Values expressed as mean (*n* = 8). * denotes *p* < 0.05, ** denotes *p* < 0.01, **** denotes *p* < 0.0001.

Figure 5 shows the effects of the both Doxil and the unloaded equivalent (Doxebo), following 24 and 48 h incubations. Both time points show a significant (*p* < 0.0001) reduction in excess of 75% when treated with the highest (200 µg/mL) concentration of Doxil. However, the 48-h data show comparable reductions at both 2 µg/mL and 20 µg/mL (*p* < 0.0001). Interestingly, the same timepoint and concentration combination (2 µg/mL and 20 µg/mL at 48 h) using the unloaded Doxebo, led to no significant changes (*p* > 0.05).

### 3.3. Cholesterol Content of Liposomes Had Little Effect on the Rate of Cell Death following Treatment

**Figure 6:**
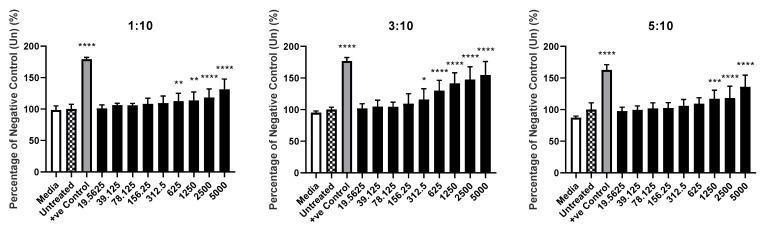
Investigation of cholesterol content and treatment concentration of materials upon cytotoxicity via LDH assay. Level of mean absorbance measured at 492 nm, compared to that of the negative control (untreated), following 24 h incubation. Concentrations stated are as ng/mL. Values expressed as mean (*n* = 8). * denotes *p* < 0.05. ** denotes *p* < 0.01. *** denotes *p* < 0.001. **** denotes *p* < 0.0001. White bar represents a cell-free, media-only control, chequered bar represents negative control (untreated), grey represents positive control, black represents treatments containing increasing concentrations of liposomes.

Across all three compositions, concentrations in excess of 625 µg/mL led to significant (*p* < 0.01) increases in cell death when compared to the negative control (untreated). The liposomes containing cholesterol at a ratio of 3:10 (CHOL:DSPC), did lead to an increase at 312.55 µg/mL, lower than either the 1:10 or 5:10 configurations (Figure 6 below).

### 3.4. Incubation of KU812 Cells with Doxil, Doxebo, or Liposome Variants Leads to Varying Levels of Histamine Release

**Figure 7:**
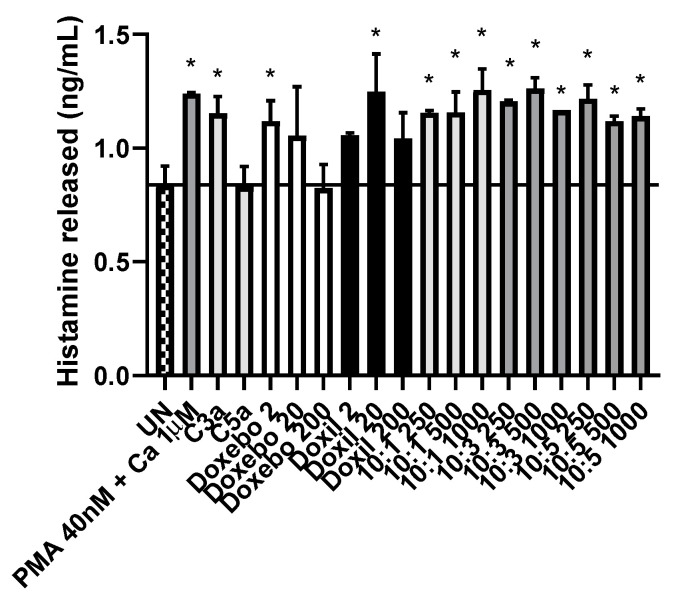
Quantity of histamine released following incubation for 24 h. Concentrations shown are stated in ng/mL unless otherwise stated. C3a and C5a were each used at a concentration 50 nM. Mean values used are calculated from *n* = 3. * denotes *p* < 0.05. Chequered bar represents a negative control (untreated), white represents unloaded Doxebo formulation, black represents loaded Doxil formulation, varying greys represents additional treatments containing materials such as PMA, calcium ionophore, C3a, C5a and liposomes.

Following incubation for 24 h, histamine release was measured via EIA. There was a significantly different response following treatment with C3a compared to C5a. Although both are key proteins within the complement cascade, C3a led to a significant increase in histamine release (*p* = 0.0177), whilst C5a showed no such influence. Treatments with either Doxil or Doxebo led to generally increased levels of histamine release, however on two of the six material-concentration combinations led to a significant difference (Doxebo [2 µg/mL], and Doxil [20 µg/mL]). Conversely however, all conditions involving liposome variants led to significant increases in histamine release (Figure 7).

### 3.5. Liposome Variants without the Addition of PEG Induced Significant IL-1β Release, from KU812 Cells, Than Those with PEG Incorporated

**Figure 8:**
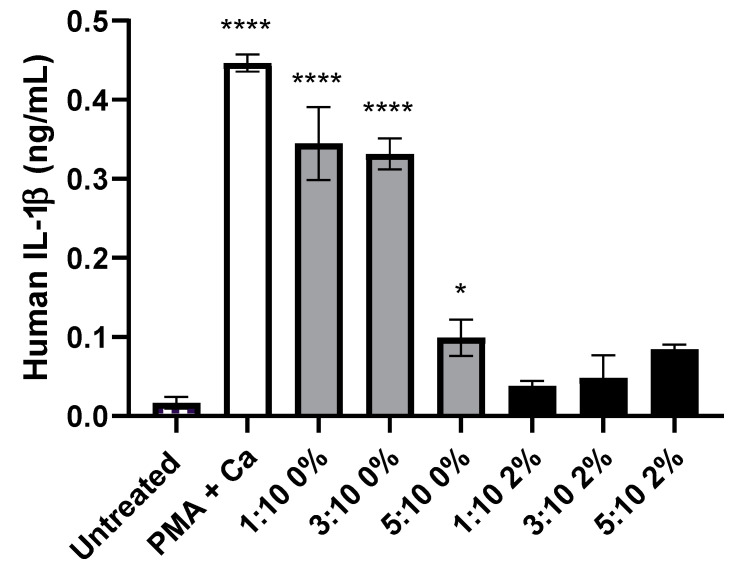
Quantity of IL-1B released by KU812 cells following incubation alongside named materials for 24 h. * denotes *p* < 0.05. **** denotes *p* < 0.0001. Chequered bar represents negative control (untreated), white represents conditions of PMA and calcium ionophore, grey representing liposomes without PEG conjugation, and black with 2% PEG conjugation.

As can be observed within Figure 8, liposomes without PEG association led to a marked, and significant (*p* < 0.05), release of Il-1β. The addition of PEG lead to a >50% reduction in IL-1β release when comparing materials containing cholesterol at a ratio of 1:10 and 3:10, whilst a smaller (5.8%) decrease was seen within the materials containing a ratio of 5:10 cholesterol. The cholesterol content of the liposomes also led to significant changes in the amount of IL-1β secreted. Within the liposomes with 0% PEG conjugation, an increase of cholesterol content led to a decrease in IL-1β, reducing over 70% from 0.33 ng/mL to 0.1 ng/mL with an increase from 3:10 to 5:10. However this correlation was reversed within the materials with 2% PEG conjugation. A >2-fold increase was seen between the values recorded for 1:10 and 5:10 ratios.

### 3.6. Distinct Basophil Activation Markers Exhibit Differing Patterns of Expression following Treatment with Liposome Variants

**Figure 9:**
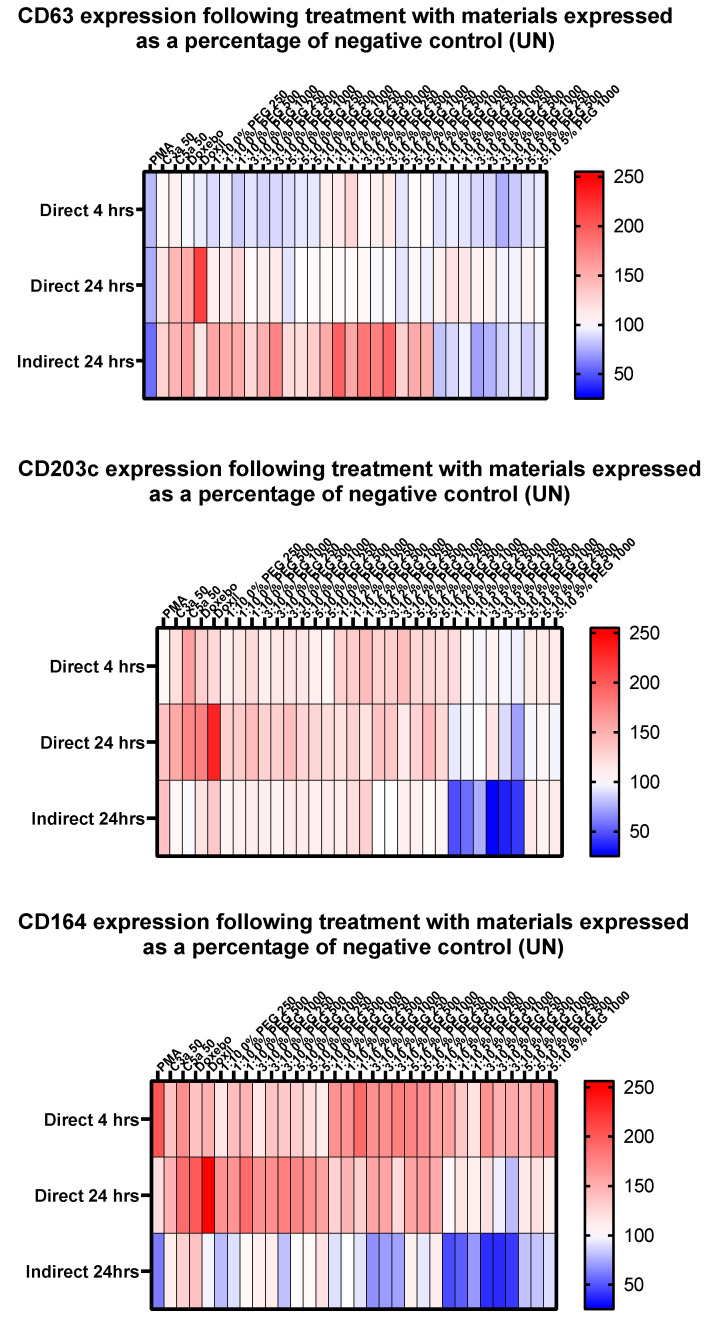
The levels of basophil activation markers, following incubation alongside various test materials. Values shown as a percentage of the negative control (Untreated). Concentrations shown are stated in ng/mL unless otherwise stated. Values shown are means calculated from *n* = 3.

As can be observed in Figure 9 (below), the three basophil activation markers CD63, CD203c and CD164 all exhibit differing responses to the range of liposomes used. CD63 expression was typically shown to be reduced following a direct incubation of 4 h, compared to 24 h where it returned to approximately the value of the negative control. Comparatively the expression after indirect incubation within pre-treated plasma was shown to increase in formulations with 0% and 2% PEG content, however it sharply declined in those with 5%.

CD203c expression was generally shown to increase across incubations of 4 and 24 h when treated directly. After 4 h, there appears to be an upregulation of expression in particular in the liposomes containing 2% PEG. Conversely, after 24 h this appears to be the case in both those liposomes with 0% or 2% PEG content, with some concentrations of 5% leading to reductions in expression when compared to the untreated negative control. Liposomes containing 5% PEG led to significant decreases when incubated within pre-treated plasma, whilst those containing 0% and 2% led to slight increases or little change from the negative control.

Figure 9 highlights that the expression of CD164 was increased following treatment with liposomes with higher PEG levels after 4 h, yet after 24 h, those with lower level of PEG led to increases. Similarly to the pattern observed with CD203c, materials with 5% PEG led to decreases in CD1654 expression following indirect treatment.

### 3.7. Association of Liposome Size, with Changes in Basophil Activation Markers

**Figure 10:**
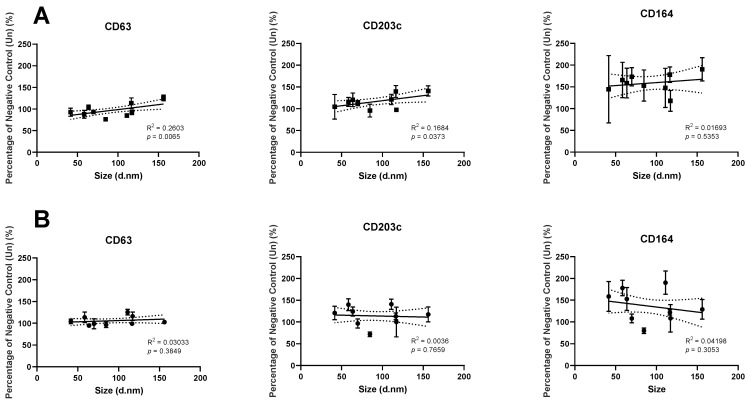
The relationship between liposome size and the resultant expression profile following incubation for 4 h (**A**) or 24 h (**B**). Mean values calculated from *n* = 3.

Analysing the liposomes used by size allows us to observe the presence of correlations between the diameter of the materials and the level of expression change. Figure 10A (4 h) features two weak, but significant, correlations in both CD63 (R^2^ = 0.03033) and CD203c (R^2^ = 0.0026), which increase with the diameter of the particles (*p* = 0.0065 and *p* = 0.0373, respectively). There were no such correlations with the data following 24 h incubation.

## 4. Discussion

The above investigation intended to use KU812 cells as a model system, to interrogate the impact of liposome variants on basophil activation profiles. Many physico-chemical characteristics are related to complement activation, so we sought to address how varying cholesterol ratios in liposome variants may also be related to the cellular component of CARPA.

The liposome variants themselves were chosen to allow for the effects of various cholesterol and PEG contents to be observed. However, through the manufacture of these materials to our desired compositions, the diameters of the liposomes were also affected, results showed that increasing cholesterol content decreased the size irrespective of PEG content. A 2% PEG addition to non-PEGylated liposomes increased the size due to long ethylene glycol unit of PEGylated lipid. It is located on the surface of liposomes. A 5% further PEG addition decreased the size again to around the non-PEGylated counterparts. This observation was in line with that of Harvie et al. [54]. Kulkarni et al. used cryo-transmission electron microscopy to show how raising PEG content affects the particle size and the result was similar with ours [55]. They hypothesized that higher PEG molarity leads to a higher surface area: volume ratio, by decreasing particle size because PEG is located around the surface of particles. Surface charge of all liposomes were neutral, but 5%PEG addition leads to more negative surface charge due to the negative feature of the Poly(ethylene glycol) chain on liposome surface.

The results of DLS analysis can be observed in Figure 3, and two distinct trends can be seen. Firstly, the higher the ratio of cholesterol to Distearoylphosphatidylcholine (DSPC), the smaller the diameter of the liposome. Those at a ratio of cholesterol-to-DSPC of 5:10 were in some cases >50% smaller than the equivalent liposome with a 1:10 ratio. Cholesterol itself has been incorporated into the production of liposomes used for drug formulation to aid with increased rigidity and stability [56,57]. Within drug formulation, cholesterol is often incorporated at a cholesterol-to-lipid ratio of 1:2, whilst the maximum that can be integrated is assumed to be 1:1 [57,58,59]. The materials used in the above study align suitably with these recommendations. Secondly, Figure 3 highlights a greater liposome size at 2% PEG, compared with either 0% or 5%. This is in line with the findings of Garbuzenko et al., who attributed the increase in size to the change in spatial structure of the PEG-lipid structure [60].

Basophil stimulation is key in the generation and release of histamine within the circulatory system [61,62]. As can be observed within Figure 7, the KU812 cell-line released significant (*p* < 0.05) histamine in response to all liposome variants used, irrelevant of the cholesterol content, except for both Avanti formulations. The latter are formed from a combination of 1,2-distearoyl-sn-glycero-3-phosphorylethanolamine (DSPE) and L-a-phosphatidylcholine (HSPC). These differences in the liposome content may be responsible for the visible differences in histamine release between the Avanti and Strathclyde materials. The variation between the levels of histamine release recorded following exposure to C3a and C5a is intriguing. Basophils are known to express C3aR and C5aR, the latter is measured to be expressed twice as frequently [63]. This level of histamine release is despite the ability of C5a to cause histamine release without the presence of IL-3, which is required for C3a trigged histamine release [64,65].

IL-1β is similar to histamine in that it is a major pro-inflammatory immunoregulatory mediator [66]. However, it is generally stimulated through the exposure of immune cells to various microbe-associated molecular patterns (MAMPs) and damage-associated molecular patterns (DAMPs) through inflammasomes such as NLRP3 [67,68,69,70]. Recently, work by Tahtinen et al. has shown that liposomes containing the ionisable cationic lipid SM-102 can induce IL-1β release from peripheral blood cells, whereas the ionisable cationic lipid MC3 is far less potent at stimulating IL-1β release [71]. It is hypothesised that this is likely via an intracellular pattern-recognition receptor such as NLRP3, however the precise mechanism by which this occurs is unclear, particularly as the liposomes may trigger other effects in the cells in which they accumulate, such as oxidative stress, or there may be peroxidation of lipids, all of which are possible inflammasome triggers but via an “indirect” activation route. The work we present here supports the findings of Tahtinen et al. [71], but demonstrating that PEGylation of the liposomes did not result in the same levels of IL-1β release compared to non-PEGylated liposomes, possibly through reduced intracellular accumulation. However, this warrants further investigation. Composition was shown to reduce the level of IL-1β released (Figure 8).

Due to the past work surrounding the cellular mechanisms of basophils, the cell surface proteins (CD63, CD203c and CD164) have been shown to have upregulated expression during immune system activation in response to allergen recognition [72,73]. Although each marker, individually, is a useful marker of the cellular state of basophils, the use of several in parallel allows for a wider scope of investigation. It is therefore useful to be able to observe the level of marker expression across a range of materials with varying characteristics. As can be seen within Figure 9, the three selected basophil activation markers exhibit patterns of expression that are distinct from one another, suggesting various routes of activation [49,73,74,75,76,77].

Figure 10 highlights the decrease in expression of CD63 by incubation with the smaller liposome variants (0% and 5%) for 4 h, whilst the 2% variants led to slight increases. The same materials recorded minimal changes from the mean following a 24 h incubation. It is feasible that this is a result of expression elevating following initial exposure to the material and then reduction via intracellular localisation or another mechanism, as such mechanisms have been shown to occur with CD63 expression upon eosinophils during granulation [78]. CD203c appeared to show an increased level of expression between the 4 and 24 h timepoints, suggesting that the pathway controlling the expression of CD203c is slower than that of CD63. A 48-h incubation timepoint would allow for this to be confirmed, as we would expect to see the trend continue, and should be considered for further study. Comparative to CD63, CD203c was seemingly stimulated greatest by the presence of 2% PEG liposomes, the largest of the liposomes used within this study. A possible explanation for larger liposomes resulting in greater activation of the KU812 cells, is that they may be better able to cross-link Fc receptors on the cell surface, a key component of basophil activation [79,80]. However, this aspect requires further investigation.

## 5. Conclusions

The data collected across this investigation support the use of multiple activation markers used within this study (CD63, CD203c and CD164) that are distinct and are activated via different pathways, instead of all being indicative of the same stimuli, and as the KU812 cell line, a useful model in the assessment of hypersensitivity responses. Each marker shows responses to varied characteristics of the liposomes used, along with the presence of known basophil activators such as C3a and C5a. Identification of the exact properties that contribute to the expression of each marker will continually increase the versatility of the KU812 cell line as both a preclinical model for liposomal nanoformulations and a tool in investigating the onset of CARPA. Further study is recommended to determine the transferability of this cell model, determination of additional secretory factors released following exposure to liposomes, such as thromboxane A2, clear definition of the mechanisms linked to activation and kinetic assessment of marker profiles to fully elucidate the cellular responses observed here.

## Figures and Tables

**Table 1:** Parameters used for DLS analysis of liposomal nanomaterials, recorded using Malvern Zetasizer Nano-S.

Material	
Refractive index	1.45
Absorption	0.001
Dispersant	PBS
Temperature	25 °C
Viscosity	1.0200 cP
Refractive index	1.335
Measurement	
Angle	173° Backscatter
Number of measurements	3

**Table 2:** Results of DLS analysis of liposomes showing mean size (d.nm) and z-potential (mV).

Molecule	Doxil^TM^	Unfilled	Liposome Formulation								
			0% PEG			2% PEG			5% PEG		
			1:10	3:10	5:10	1:10	3:10	5:10	1:10	3:10	5:10
Mean size d.nm (*n* = 3)	120	115	111.0	58.38	41.5	156.2	116.5	63.68	117.2	84.73	69.79
Mean z-Potential mV (*n* = 3)	−0.42	−0.60	−0.51	−0.84	−0.42	−0.82	−0.05	1.11	−2.08	−1.20	−2.28

## Data Availability

Data available on request due to privacy reasons. The data presented in this study are available on request from the corresponding author. The data are not publicly available as they may contain sensitive information.

## Abbreviations

Polyethylene glycol (**PEG**), Complement-activation related pseudoallergy (**CARPA**), Nanoparticle (**NP**), Phorbol-12-myristate-13-acetate (**PMA**), Calcium ionophore (**CA**), Hypersensitivity reaction (**HSR**), Roswell park memorial institute media (**RPMI**), Foetal bovine serum (**FBS**), Inactive complement 3b (**IC3b**), Cobra venom factor (**CVF**), 3-(4,5-dimethylthiazol-2-yl)-2,5-diphenyltetrazolium bromide (**MTT**), Dimethyl sulfoxide (**DMSO**), Toll-like receptor (**TLR**).
